# A multiplex endpoint RT-PCR assay for quality assessment of RNA extracted from formalin-fixed paraffin-embedded tissues

**DOI:** 10.1186/1472-6750-10-89

**Published:** 2010-12-17

**Authors:** Elena A Takano, Thomas Mikeska, Alexander Dobrovic, David J Byrne, Stephen B Fox

**Affiliations:** 1 Molecular Pathology Research and Development Laboratory, Department of Pathology, Peter MacCallum Cancer Centre, East Melbourne, Victoria 3002, Australia; 2 Department of Pathology, The University of Melbourne, Parkville, Victoria 3010, Australia

## Abstract

**Background:**

RNA extracted from formalin-fixed paraffin-embedded (FFPE) samples is chemically modified and degraded, which compromises its use in gene expression studies. Most of the current approaches for RNA quality assessment are not suitable for FFPE derived RNA.

**Results:**

We have developed a single-tube multiplex endpoint RT-PCR assay specifically designed to evaluate RNA extracted from FFPE tissues for mRNA integrity and performance in reverse transcription - quantitative real-time PCR (RT-qPCR) assays. This single-tube quality control (QC) assay minimises the amount of RNA used in quality control. mRNA integrity and the suitability of RNA for RT-PCR is evaluated by the multiplex endpoint RT-PCR assay using the *TBP *gene mRNA as the target sequence. The RT-PCR amplicon sizes, 92, 161, 252 and 300 bp, cover a range of amplicon sizes suitable for a wide range of RT-qPCR assays. The QC assay was used to evaluate RNA prepared by two different protocols for extracting total RNA from needle microdissected FFPE breast tumour samples. The amplification products were analysed by gel electrophoresis where the spectrum of amplicon sizes indicated the level of RNA degradation and thus the suitability of the RNA for PCR. The ability of the multiplex endpoint RT-PCR QC assay to identify FFPE samples with an adequate RNA quality was validated by examining the C_q _values of an RT-qPCR assay with an 87 bp amplicon.

**Conclusions:**

The multiplex endpoint RT-PCR assay is well suited for the determination of the quality of FFPE derived RNAs, to identify which RT-PCR assays they are suitable for, and is also applicable to assess non-FFPE RNA for gene expression studies. Furthermore, the assay can also be used for the evaluation of RNA extraction protocols from FFPE samples.

## Background

Formalin-fixed paraffin-embedded (FFPE) tissue samples are routinely used for diagnosis of disease. There is increasing interest in extracting RNA from these samples as the large numbers of archival FFPE samples constitute an invaluable resource for the investigation of diagnostic, prognostic or predictive disease associated alterations in gene expression (reviewed in [1]). Furthermore, these often represent the only diagnostic material available.

However, RNA extracted from FFPE specimens is extensively degraded and chemically modified, which compromises its use in PCR based applications (reviewed in [2]). Degradation of RNA is influenced by the time and storage conditions between sample collection and formalin fixation, the fixation process, and the conditions and length of the subsequent storage [3]. Chemical modifications of RNA are caused by formaldehyde and result in the addition of mono-methylol groups to RNA bases and subsequently in the formation of methylene bridges between RNA bases [4], and RNA-protein cross-links [5]. The addition of the mono-methylol group is in principal reversible, but a considerable amount is still present after RNA purification [4].

Whereas the extent of chemical modifications of the RNA initially limits the PCR amplification size, with time RNA degradation becomes more important in determining the size of amplifiable PCR fragments [3]. The RNA fragment sizes from FFPE tissue are usually less than 300 bp and may be less than 100 bp [3,6-8].

The quality of the extracted RNA is a critical factor for both microarray based and reverse transcription - quantitative real-time PCR (RT-qPCR) gene expression experiments. Microarray based approaches are more sensitive to RNA degradation and chemical modifications [2], which potentially influence the gene expression data [9].

RT-qPCR assays are less affected by RNA degradation and chemical modifications especially when the RT-qPCR amplicon size is less than 100 bp [10]. It is nevertheless an essential prerequisite to characterise the quality of the extracted RNA prior to its use in a gene expression study to evaluate its suitability for the planned application and to minimise data misinterpretation [11,12].

RNA quality is often defined in terms of RNA purity and RNA integrity. RNA purity is spectrophotometrically determined by the A_260_/A_280 _and A_260_/A_230 _absorbance ratios [13-15]. Both ratios are used to evaluate the level of contaminants such as proteins and residual organic compounds present in a RNA sample. These values provide no information about RNA degradation and amplifiable PCR amplicon sizes.

RNA integrity evaluates the level of RNA degradation and several methods have been developed for RNA integrity assessment. Two of the most commonly used approaches are suitable for assessing moderately degraded RNA but not highly degraded RNA.

The first approach investigates the ratio between the 28S and 18S ribosomal RNA bands and presumes that the integrity of ribosomal RNA reflects the integrity of mRNA [16,17]. The typical RNA fragment sizes of less than 300 bp make this methodology unsuitable for highly degraded RNA from FFPE samples [8]. Furthermore, this approach does not take chemical modifications of the RNA into account and provides no information on how the extracted RNA performs in RT-PCR.

The second approach has been adopted from a common practice in microarray experiments [18] and determines the mRNA integrity by utilising RT-qPCR assays to assess the 3':5' ratio of a gene target sequence, such as *GAPDH *[19] or *ACTB *[12]. These assays need to reach a considerable fragment size (up to 1.2 kb) to calculate the 3':5' ratio, which makes this methodology unsuitable for highly degraded RNA from FFPE samples. Moreover, the 3':5' assays utilise an oligo-dT primer for cDNA preparation, which precludes the cDNA obtained from being used in the majority of RT-PCR assays, an important factor when the amount of RNA is often limited.

The third approach assesses a range of fragment sizes generated by (multiplexed) endpoint RT-PCR assays for certain reference (housekeeping) genes, such as *G6PD*, *TBP*, *HPRT *and *ACTB *[3,20-22]. This approach takes both fragmentation and chemical modifications of the RNA into account and determines the PCR amplicon sizes, which might be obtained in a PCR based application. When the PCR amplicon sizes cover a range from less than 100 bp to several hundred base pairs, this methodology is suitable to assess the extent of the degradation of RNA extracted from FFPE tissues.

In this study, we sought to combine the best features of assays from the third approach to develop a readily performable endpoint RT-PCR assay to assess RNA extracted from FFPE samples for mRNA integrity and RNA performance in RT-qPCR assays.

The assay utilises the *TBP *(TATA box binding protein) reference gene mRNA as the target sequence. *TBP *has been shown to be relatively stably expressed in a range of tissues [23] and various tumour types (e.g. [24-29]). Four different amplicon sizes are amplified in parallel in a single tube from cDNA (multiplexed endpoint RT-PCR assay) to minimise the consumption of what are often limited amounts of RNA. The amplicon sizes chosen cover a range of up to 300 bp and are therefore tailored to the fragment size limitations typically observed for RNA extracted from FFPE samples and PCR amplicon sizes usually used for the vast majority of RT-qPCR assays.

Our improved multiplex endpoint RT-PCR assay is a robust and convenient method which overcomes the limitations of current approaches for the quality assessment of RNA extracted from FFPE specimens. The assay has been thoroughly validated by assessing the quality of 180 RNA samples extracted from FFPE tissues with an RT-qPCR assay.

## Results

### Assessment of total RNA yield and purity using optical density

**Figure 1:**
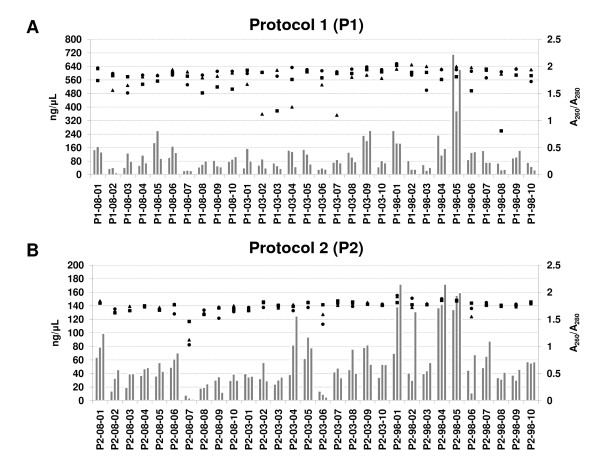
**Assessment of total RNA yield and purity extracted by the two different RNA extraction protocols**. The x-axis shows the sample identification. The y-axis on the left shows the RNA concentration in ng/μL. The RNA concentrations for each of the sample replicates are shown as groups of three bars. The y-axis on the right shows the absorbance ratio A_260_/A_280_. The absorbance ratio A_260_/A_280 _for each of the sample replicates is shown as a scatter graph (■: replicate 1; ▲: replicate 2; ●: replicate 3). Panel A corresponds to protocol 1 (P1) and Panel B corresponds to protocol 2 (P2). Note that for the RNA concentration, panel A is scaled differently to panel B as the RNA extracted by the two different RNA extraction protocols was eluted in different final elution volumes.

The amount of total RNA extracted for each of the samples was measured by a NanoDrop ND-2000 spectrophotometer and is shown for each sample replicate in Figure 1. The estimated mean total RNA extracted for protocol 1 was 2.23 μg (111.5 ng/μL) with values ranging from 0.24 to 14.24 μg (12.0 to 712.0 ng/μL). For protocol 2, the estimated mean concentration was 2.76 μg (55.1 ng/μL) with values ranging from 0.12 to 3.61 μg (2.4 to 72.1 ng/μL).

The corresponding absorbance ratio A_260_/A_280 _for each sample replicate is shown in Figure 1. The mean A_260_/A_280 _ratio for protocol 1 was 1.8 ± 0.2 and for protocol 2 1.7 ± 0.1. The desired ratio A_260_/A_280 _is in the range of 1.7 to 2.1 and is dependent on the extraction conditions [30].

### The use of the multiplex endpoint RT-PCR for assessment of mRNA integrity

**Figure 2:**
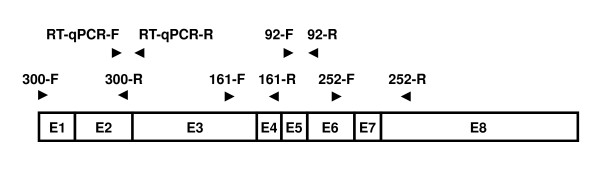
**Primer placement for the multiplex endpoint RT-PCR and the RT-qPCR assay**. Both assays use the *TBP *mRNA (NM_003194) as the target sequence. The eight exons are shown as rectangles labelled from E1 to E8. The primer locations are shown as horizontal arrow heads. The positions of the primers for the multiplex endpoint RT-PCR assay (92-F, 92-R, 161-F, 161-R, 252-F, 252-R, 300-F and 300-R) and for the RT-qPCR assay (RT-qPCR-F and RT-qPCR-R) are shown. Each primer pair was designed to span at least one intron.

**Figure 3:**
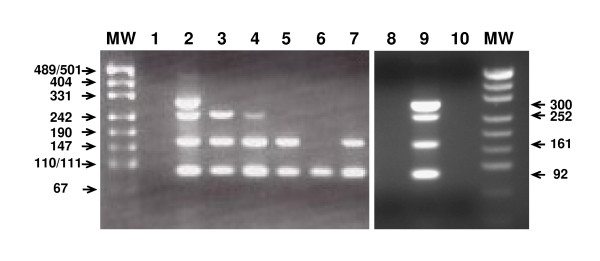
**Assessment of RNA degradation and RNA performance in RT-PCR by the multiplex endpoint RT-PCR assay**. The sizes of the molecular weight markers (MW) are given on the left, whereas the sizes of the *TBP *amplicons are indicated on the right. Sizes are given in base pairs. Lane 1 and 10 were loaded with the no template control (NTC). Lane 2 and 9 were loaded with the PCR reaction obtained from a cDNA mixture synthesised from RNA extracted from different cell lines and serves as positive control, showing all four PCR amplification products of the expected size. Lane 8 was loaded with the PCR reaction obtained from genomic DNA and served as negative control. Lanes 3 to 7 were loaded with RT-PCR reactions obtained from cDNAs synthesised from total RNA derived from needle microdissected FFPE breast tumour tissues (Lane 3: P1-98-05, replicate 1; Lane 4: P1-98-06, replicate 1; Lane 5: P1-98-07, replicate 1; Lane 6: P1-98-08, replicate 1; Lane 7: P1-98-09, replicate 1).

The integrity of the mRNA extracted from each sample was assessed by the multiplex endpoint RT-PCR assay using the *TBP *(TATA box binding protein) mRNA (NM_003194) as the target sequence (Figure 2). The assay was designed to amplify four amplicons of 92, 161, 252 and 300 bp in parallel in a single tube (Figure 3).

The amplicons were designed to be amplified from cDNA but not genomic DNA (Figure 3). This is of particular importance for assessing cDNA synthesised from total RNA, which has not been treated with DNase. We used primer pairs where the primers were in exons spanning one or more introns (Figure 2). Furthermore, the primer locations were chosen to exclude known polymorphic sites from the primer binding sites.

**Figure 4:**
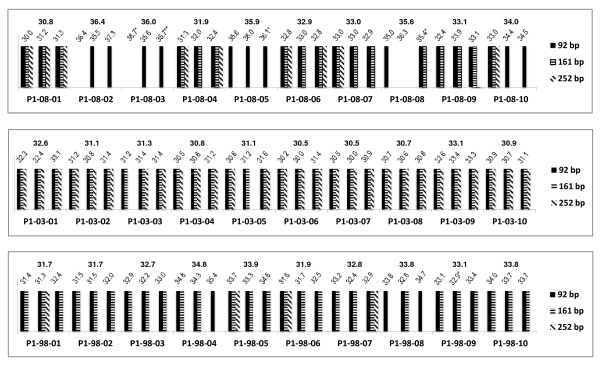
**Comparison of the multiplex endpoint RT-PCR and RT-qPCR assays for protocol 1 (P1)**. The bottom axis identifies the samples. The band sizes obtained for each of the sample replicate are shown as bars. The values on the top of the graph provide both the C_q _value measured for each sample replicate in the *TBP *RT-qPCR assay and the mean C_q _value. Symbols are as follows: * indicates a standard deviation between replicates that is greater than 0.5; ** one out of two replicates amplified.

**Figure 5:**
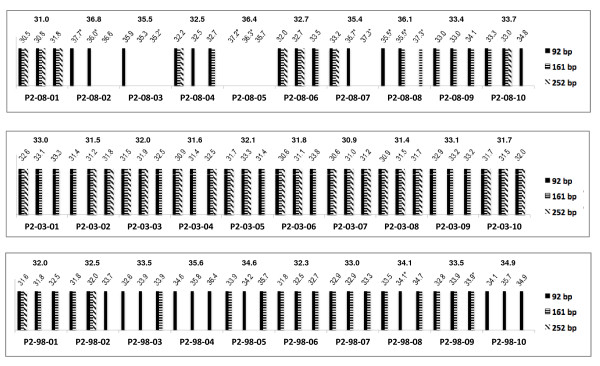
**Comparison of the multiplex endpoint RT-PCR and RT-qPCR assays for protocol 2 (P2)**. The bottom axis identifies the samples. The band sizes obtained for each of the sample replicate are shown as bars. The values on the top of the graph provide both the C_q _value measured for each sample replicate in the *TBP *RT-qPCR assay and the mean C_q _value. Samples labelled with a * symbol indicate a standard deviation between replicates that is greater than 0.5.

The results for protocols 1 and 2 are summarised in Figures 4 and 5 for each sample replicate. In general, the least degraded RNA was extracted from the year 2003 samples for both protocols (Figures 4 and 5). In addition, the vast majority of sample replicates of both protocols amplified the 161 bp and 252 bp PCR fragments (Figures 4 and 5, Table 1).

**Table 1:** Correlation of the multiplex endpoint RT-PCR and the RT-qPCR assay.

Multiplex RT-PCR	Protocol	No. samples in RT-PCR	No. samples in RT-qPCR
No amplification	P1	3/90 (3%)	Cq < 35.1 : 0 Cq ≥ 35.1 : 3
	P2	7/90 (8%)	Cq < 35.1 : 0 Cq ≥ 35.1 : 7
92 bp	P1	13/90 (15%)	Cq < 35.1 : 5 Cq ≥ 35.1 : 8
	P2	18/90 (20%)	Cq < 35.1 : 10 Cq ≥ 35.1 : 8
>92 bp	P1	74/90 (82%)	Cq < 35.1 : 73 Cq ≥ 35.1 : 1
	P2	65/90 (72%)	Cq < 35.1 : 62 Cq ≥ 35.1 : 3

### Assessment of mRNA performance by RT-qPCR

The performance of the mRNA extracted from each sample was assessed by a RT-qPCR assay also using the *TBP *mRNA as the target sequence (Figure 2). The RT-qPCR assay was designed and optimised to be monitored using hydrolysis probes from the Universal Probe Library (UPL) library in combination with gene-specific primers [31,32]. The assay amplifies an amplicon of 87 bp from cDNA using an intron-spanning primer pair. The quantification cycle (C_q_) [33] values obtained for each sample replicate in the RT-qPCR assay were used to estimate the quantity of amplifiable template.

Each sample replicate was classified into one of three different groups based on the C_q _values measured (C_q _≤ 32.0; 32.1 ≤ C_q _≤ 35.0; 35.1 ≤ C_q _≤ 39.9) (Table 2). The C_q _value is inversely proportional to the number of amplifiable templates. A C_q _value of 35 is generally considered as the limit for the detection of a single copy template [34,35]. A C_q _value above 35 thus represents less than one copy template present and can be considered as background noise of the RT-qPCR assay [35]. A theoretical C_q _value of 32 represents approximately 10 copies of the target template.

**Table 2:** Cq values measured by the RT-qPCR assay.

	**C**_**q **_**≤ 32**	**32.1 ≤ C**_**q **_**≤ 35.0**	**35.1 ≤ C**_**q **_**≤ 39.9**
P1-08	5	14	11
P1-03	24	6	0
P1-98	7	22	1
**P1 (total)**	**36**	**42**	**12**
			
P2-08	4	12	14
P2-03	20	10	0
P2-98	5	21	4
**P2 (total)**	**29**	**43**	**18**

Samples from years 2008, 2003 and 1998 were analysed by each of the two protocols. Each sample of protocol 1 (P1) and 2 (P2) was separated into three groups (based on the C_q _value measured for each sample replicate).

Eighty seven percent (78/90) of samples extracted by protocol 1 and 80% (72/90) of samples isolated by protocol 2 showed C_q _values less than 35.1 for the *TBP *RT-qPCR assay (Figures 4 and 5, Table 2). Therefore, cDNA prepared from RNA extracted with protocol 1 might had slightly more amplifiable templates per sample volume than cDNA prepared from total RNA extracted with protocol 2.

### Comparison of the multiplex endpoint RT-PCR and RT-qPCR assays

The results from the multiplex endpoint RT-PCR assay were validated by the results obtained from the RT-qPCR assay and allowed the identification of FFPE samples with an adequate RNA quality.

Almost all the samples (135/139) that amplified the PCR fragments more than 92 bp in the multiplex endpoint RT-PCR assay amplified the 87 bp product at C_q _values less than 35.1 in the RT-qPCR assay consistent with a greater amount of amplifiable template (Figures 4 and 5, Table 1).

Six percent (10/180) of the samples did not amplify in the multiplex endpoint RT-PCR assay (Table 1). Consistent with this, the absence of the 92 bp amplicon was associated with late amplification in the RT-qPCR assay with a C_q _value later than 35.0 (Figures 4 and 5, Table 1).

In some cases, stochastic effects due to the very small number of available templates, can give inconsistent results; e.g. for one of the replicates of sample P2-08-08, the 92 bp amplicon did not amplify in the multiplex endpoint RT-PCR assay but showed a band for the 161 bp product (Figure 5). The other replicates showed a 92 bp band and band sizes up to the 161 bp amplicon, respectively. All three replicates showed C_q _values in the RT-qPCR assay above 35.0 (Figure 5) indicating that on average less than one copy of template was present.

We saw no FFPE samples that gave robust amplification for the 300 bp amplicon. Forty six percent (41/90) of the samples prepared by protocol 1 amplified all the amplicons up to 252 bp in the multiplex endpoint RT-PCR assay (Figure 4). These samples showed a mean C_q _value in the RT-qPCR assay of 31.5 ± 1.0 with values ranging from 30.0 to 33.7. For protocol 2, 33% (30/90) of the samples amplified the amplicons up to 252 bp and showed a mean C_q _value of 31.7 ± 0.8 with values ranging from 31.7 to 33.3 (Figure 5).

**Figure 6:**
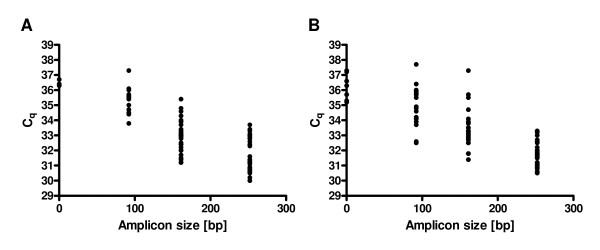
**Spearman correlation between fragment size in the multiplex endpoint RT-PCR assay and the appropriate C_q _value obtained in the RT-qPCR assay**. The x-axis shows the fragment size and the y-axis shows the C_q _value. Panel A corresponds to protocol 1 (P1) and Panel B corresponds to protocol 2 (P2).

Correlations between a longer amplicon size in the multiplex endpoint RT-PCR assay and the appropriate C_q _value obtained in RT-qPCR were evaluated by calculating the Spearman correlation coefficient. We found an inverse correlation for both parameters for protocols 1 and 2 [*r*(P1) = -0.75, CI (95%) = -0.83 to -0.65 (*P *< 0.0001, n = 90) and *r*(P2) = -0.80, CI (95%) = -0.86 to -0.70 (*P *< 0.0001, n = 90), respectively]. Thus, a longer fragment size in the multiplex endpoint RT-PCR assay was correlated with a smaller C_q _value in the RT-qPCR assay for both total RNA extraction protocols (Figure 6).

Interestingly, both the multiplex endpoint RT-PCR and the RT-qPCR assay show that the samples prepared from 2003 performed best during PCR amplification. All 60 samples from 2003 extracted by protocols 1 and 2 amplified PCR amplicons up to 161 bp in the multiplex endpoint RT-PCR assay (Figures 4 and 5). Furthermore, all of these samples also amplified the 87 bp amplicon with C_q _values less than 35.1 in the RT-qPCR assay (Figures 4 and 5). The lack of correlation between age of the FFPE samples and RNA quality has been reported previously [36]. This may be explained by a difference in the processing of the samples, such as pre-fixation time, fixation time and storage conditions over the course of time [3,21]. The variation in PCR fragment sizes present in each sample replicate was also the least in the samples prepared in year 2003 compared to samples prepared from the years 1998 and 2008, consistent with a minimisation of stochastic effects caused by greater amounts of template. The effect of sample age needs further investigation.

## Discussion

There has been an increasing demand for reliable methods and protocols for the extraction of RNA from FFPE tissue sections. The performance of RNA preparation protocols has improved over the years and specific protocols for certain RNA downstream applications have been developed (reviewed in [1,2]).

Despite the improvements in methodologies for extracting RNA from FFPE specimens, one significant and challenging problem still remains. The quality of the extracted RNA is compromised and the degree of RNA degradation and the extent of chemical modifications of the RNA may limit its use in downstream applications. It is therefore critical to assess the RNA quality at the cDNA stage to identify the RNA preparations which are suitable for a particular RT-qPCR analysis.

The multiplex endpoint RT-PCR approach used in this study has been developed and optimised to assess RNA extracted from FFPE specimens for its use in RT-qPCR assays. The assay is sensitive to RNA degradation as well as chemical modifications, which both determine the length of an amplicon during PCR amplification. This methodology is similar in principle to one that has been used extensively in our laboratory for the assessment of DNA from FFPE samples [37].

The choice of the target gene mRNA is of particular importance. It is desirable, that the target gene mRNA is ubiquitously expressed across most cell types and that the level of gene expression shows a similar magnitude among the samples. We selected *TBP *for our multiplex endpoint RT-PCR assay, because it was shown to be relatively stably expressed in a range of tumour types such as bladder cancer [24], renal cell carcinoma [25], hepatocellular carcinoma [26], glioma [27,29] and breast cancer [28]. *TBP *is expressed at moderate [27,29,38] to low levels [23]. The choice of a highly expressed target gene mRNA might result in an increased false negative rate for less abundant mRNAs in gene expression studies, due to an insufficient amount of starting material for PCR amplification. This is supported by a study, which showed that only a limited proportion of the RNA extracted from FFPE tissues is actually accessible for cDNA synthesis [39].

We also designed our primers to avoid underlying polymorphic sites. Target gene mRNA from highly polymorphic genes perhaps should be avoided. The primers in a previous approach which used the *G6PD *gene actually overlie potential polymorphic sites [20]. Primer binding sites which contain polymorphic sites will lead to impaired or even absent PCR amplification of the mismatched alleles.

Our multiplex endpoint RT-PCR approach utilises random primers to synthesise cDNA. Thus after the cDNA is assessed, the same cDNA can subsequently be used in the final gene expression experiment(s). This then also controls for the cDNA synthesis step.

The knowledge about which fragment sizes can be amplified during a RT-qPCR assay helps to identify samples suitable for gene expression analysis and which amplicon sizes can be used for an RT-qPCR assay design. The range of PCR amplicon sizes covered by our assay takes into account the RNA sizes normally obtained from FFPE. The upper size range is interrogated by three PCR amplicon sizes (161, 252 and 300 bp), and helps to identify samples suitable for sometimes more demanding applications, such as discrimination between gene splice variants. The 92 bp PCR fragment helps to identify samples suitable for FFPE-friendly RT-qPCR assays which we normally design with amplicon sizes less than 90 bp.

The good agreement between the results observed for the multiplex endpoint RT-PCR assay and the RT-qPCR results (Figure 6) shows that this assay can identify FFPE samples suitable for gene expression studies. The ten samples, which did not amplify in the multiplex endpoint RT-PCR assay amplified all very late in the RT-qPCR assay and would normally have been excluded from further analysis. The 71 samples, which showed amplification of all the amplicon sizes up to 252 bp in the multiplex endpoint RT-PCR assay amplified with moderate C_q _values in the RT-qPCR assay and are probably the best suited samples to deliver reliable gene expression data for most genes of interest in gene expression analysis.

## Conclusions

There is an increasing demand for the use of RNA from FFPE both in research and in molecular diagnostic applications such as the Onco*type *DX test [6,40,41]. This is accompanied by a need for reliable methodologies to assess the quality of FFPE derived RNA. Most of the approaches currently used for RNA quality control are not suitable or have a limited usage for the assessment of such challenging material.

The validated quality control multiplex endpoint RT-PCR assay presented here overcomes the limitations of current approaches and is a robust method and well suited for determining the quality of a RNA preparation, especially for FFPE derived RNA. In addition, the assay can be used for routine quality control assessment of cDNA synthesis. The assay is also applicable for comparing or refining methodologies for RNA extraction and cDNA synthesis. Finally, the approach is cost effective and only requires equipment which is widely available.

## Methods

### Archival FFPE tissue samples

Thirty breast tumour FFPE blocks (ten each from the years 1998, 2003 and 2008) were retrieved from the archives of the Department of Pathology at the Peter MacCallum Cancer Centre. The institutional ethics committee approved the study (Approval number: 03/90).

### Histology procedures

The bench surface, the manual rotary microtome Leica RM2235 (Leica Microsystems), the equipments and glass slides were cleaned with RNaseZap (Ambion, Life Technologies, Austin, TX) prior to use according to the manufacturer's directions. Diethyl pyrocarbonate (DEPC) (Sigma, St. Louis, MO) treated water (0.1%, v/v) was used throughout the histology procedures.

For haematoxylin and eosin (H&E) staining, a 3 μm section from each FFPE block was stained to identify the tumour enriched area for needle microdissection. From each FFPE block, one to five 7 μm sections were prepared and mounted on the glass slides. The number of sections used in microdissection was determined by the content of tumour cells in the tumour enriched marked areas therefore ensuring a adequate amount of tumour cells to be microdissected for each sample. The content of tumour cells in all cases was assessed by a pathologist and was in the range of 40 to 90%. After baking the sections for five minutes on a hotplate at 70°C, the sections were deparaffinised in three changes of xylene for two minutes each and were taken to water by three changes of 100% ethanol for two minutes each and DEPC water for two minutes. Subsequently, the sections were stained with 0.5% methyl green to assist with needle microdissection. FFPE blocks were sectioned freshly just prior to needle microdissection and subsequent total RNA extraction to minimise RNA degradation after sectioning.

### RNA extraction and complementary DNA (cDNA) preparation

Two protocols were used to extract total RNA from FFPE samples. The first protocol utilised the High Pure FFPE RNA Micro Kit (Roche, Mannheim, Germany) and the second protocol utilised TRIzol reagent. The latter protocol was adapted as described from a recently published protocol [8]. The experimental procedures were performed in triplicates, each single experiment on a different day.

In the first protocol referred to as protocol 1 (P1), needle microdissected tissue from each sample was transferred immediately to a 1.5 mL Eppendorf tube containing 60 μL tissue lysis buffer provided by the High Pure FFPE RNA Micro Kit. Total RNA was extracted according to the manufacturer's protocol, eluted in 20 μL elution buffer and stored at -80°C.

In the second protocol referred to as protocol 2 (P2), needle microdissected tissue from each sample was immediately transferred to a 1.5 mL Eppendorf tube containing 260 μL of the lysis buffer from the Agencourt FormaPure Kit (Beckman Coulter, Beverly, MA) and incubated at 70°C for one hour. Subsequently the tube was cooled to room temperature, 20 μL proteinase K provided with the FormaPure Kit was added and incubated at 55°C for one hour. The reaction mixture was cooled to room temperature, 500 μL TRIzol (Gibco BRL, Life Technologies, Grand Island, NY) and 100 μL chloroform were added, vortexed thoroughly and incubated for three minutes. After centrifugation of the tube at 12,000*g *for 14 minutes at 4°C the upper aqueous layer was immediately transferred into a new 1.5 mL Eppendorf tube. An equal volume of 2-propanol was added to the aqueous layer, vortexed thoroughly and incubated overnight at -20°C. The sample was centrifuged at 12,000*g *for 10 minutes at 4°C. The supernatant was removed. The RNA pellet was washed with 1 mL 70% ethanol (4°C), vortexed thoroughly and centrifuged at 12,000*g *for five minutes at 4°C. The supernatant was removed and the 70% ethanol washing step was repeated. The supernatant was removed again and the RNA pellet was dried for 15 minutes at room temperature and resuspended in 30 μL RNase free water. Residual genomic DNA co-extracted with total RNA was digested by DNase treatment employing the Turbo DNA-Free kit (Ambion) according to the manufacturer's instructions in a final volume of 50 μL. The extracted total RNA was stored at -80°C.

Total RNA concentrations were measured by the NanoDrop ND-2000 spectrophotometer (NanoDrop Technologies, Thermo Fisher Scientific, Wilmington, DE) after DNase treatment for each sample derived by each protocol in duplicate. Mean values were used to calculate the total RNA input for cDNA synthesis. RNA purity was estimated by the absorbance ratio A_260_/A_280_.

One hundred fifty and 75 ng respectively of total RNA was reverse transcribed using the Superscript III Reverse Transcriptase (Invitrogen, Life Technologies, Carlsbad, CA) with 250 ng random hexamers (Pharmacia, Uppsala, Sweden) according to the manufacturer's instructions in the presence of 20U RNase inhibitor (Roche) in a final volume of 20 μL. The mixture was incubated for one hour at 55°C. The resulting cDNA was stored at -20°C.

### Multiplex endpoint RT-PCR

Presence or absence of various cDNA fragments synthesized from RNA extracted from the FFPE samples was determined by a multiplex endpoint RT-PCR assay using the *TBP *mRNA (NM_003194) as the target sequence (Figure 2). The assay conditions were optimised on a cDNA mixture prepared from RNA extracted from different cell lines.

PCR was performed on the Veriti 96-well Thermal Cycler (Applied Biosystems, Life Technologies, Foster City, CA) in a Thermo-Fast 96 PCR Detection Plate MkII (ABgene, Thermo Fisher Scientific, Epsom, United Kingdom) with a final reaction volume of 20 μL, containing 350 nmol/L each of the primers 92-F: 5'-GGATAAGAGAGCCACGAACC-3' and 92-R: 5'-TGCCAGTCTGGACTGTTCTT-3', 550 nmol/L each of the primers 161-F: 5'-GGGCACCACTCCACTGTAT-3' and 161-R: 5'-CACGAAGTGCAATGGTCTTT-3', 250 nmol/L each of the primers 252-F: 5'-GGGAGCTGTGATGTGAAGTTT-3' and 252-R: 5'-TGAGAGCCATTACGTCGTCT-3', 100 nmol/L each of the primers 300-F: 5'-GGCGGAAGTGACATTATCAA-3' and 300-R: 5'-CAGGCTGTTGTTCTGATCCA-3' (GeneWorks, Adelaide, Australia), 200 μmol/L of each dNTP, 0.5U of HotStarTaq DNA Polymerase (Qiagen, Hilden, Germany) in 1× of the supplied PCR buffer containing 2.5 mmol/L MgCl_2 _and 1 μL (if 150 ng of total RNA was used for cDNA preparation) and 2 μL (if 75 ng of total RNA was used for cDNA preparation) of undiluted cDNA as template respectively. The initial denaturation (95°C, 15 minutes) was followed by 40 cycles of 30 seconds at 95°C, 30 seconds at 60°C and 30 seconds at 72°C, and a final extension step at 72°C for seven minutes. Genomic DNA extracted from peripheral blood from normal individuals (2 ng/μL) was used as negative control and a cDNA mixture prepared from total RNA of different cell lines served as a positive control. Each sample was analysed once.

The PCR products were evaluated for band abundance and size by agarose gel electrophoresis. The samples were run on a 2% (w/v) agarose gel in a 1× TBE Buffer system and stained with ethidium bromide. The wells were loaded with 20 μL of the PCR product mixture with 5 μL 5× loading dye. One μL pUC19/*HpaII *DNA Molecular Weight Marker (GeneWorks) was run alongside the PCR products to determine their size.

### Reverse transcription - quantitative real-time PCR (RT-qPCR)

PCR was performed on the LightCycler 480 Instrument (Roche). Resulting data were analysed and quantified with the LightCycler 480 software release 1.5.0 (Roche), utilising the second derivative maximum method [42]. The calculated C_P _(Crossing Point) value corresponds to the current recommended term C_q _(Quantification Cycle) value [33].

PCR was performed in LightCycler 480 Multiwell Plate 384 plates (Roche) in a final reaction volume of 10 μL using the *TBP *mRNA (NM_003194) as the target sequence (Figure 2). The *TBP *RT-qPCR assay was designed and optimised as described previously [32]. 300 nmol/L of the forward primer RT-qPCR-F: 5'-GAACATCATGGATCAGAACAACA-3' and 200 nmol/L of the reverse primer RT-qPCR-R: 5'-ATAGGGATTCCGGGAGTCAT-3' (GeneWorks) were mixed in 1× LightCycler 480 Probes Master (Roche) containing 100 nmol/L of the human Universal Probe Library probe #87 (Roche), and 1 μL (if 150 ng of total RNA was used for cDNA preparation) or 2 μL (if 75 ng of total RNA was used for cDNA preparation) of undiluted cDNA as template respectively. The initial denaturation (95°C, 10 minutes) was followed by 45 cycles of 10 seconds at 95°C, 30 seconds at 60°C, and a final cooling step at 40°C for 30 seconds. Each sample was analysed in duplicate.

### Statistical analysis

Statistical analyses were performed using GraphPad Prism version 5.03 for Windows (GraphPad Software, San Diego, CA, http://www.graphpad.com). Where appropriate, data are presented as the mean ± standard deviation (SD). Nonparametric correlations between a longer amplicon size in the multiplex endpoint RT-PCR assay and the appropriate C_q _value obtained in RT-qPCR were evaluated by calculating the Spearman correlation coefficient. A two-tailed *P*-value (calculated by Gaussian approximation) of <0.05 was considered to be statistically significant for each correlation.

## Competing interests

The authors declare that they have no competing interests.

## Authors' contributions

EAT, TM and DJB carried out the experiments. TM, EAT and AD conceived the experiments and analysed the data. SBF, AD and TM initiated the project and supervised the work. All authors contributed important ideas throughout the project and were involved in the writing of the manuscript. All authors have read and approved the manuscript.
